# Editorial: Medical Physicists as Educators

**DOI:** 10.1120/jacmp.v10i3.3147

**Published:** 2009-07-21

Just over a year ago I was fortunate to have attended a workshop entitled “Becoming a better teacher of medical physics.” The workshop was sponsored by the American Association of Physicists in Medicine and was targeted at medical physicists who are involved in teaching, whether it be to medical physics students, medical residents, technologists, or any other population group. Presenters included medical physicists, physicians, and physics educators, and much of the workshop involved active participation by the physicists in attendance.

As a medical physicist who is heavily involved in the teaching of medical physics to medical residents, as well as to medical physics graduate students, I learned several lessons from the workshop. In particular, I became aware of what I believe to be a problem facing medical physicists as educators, and an explanation of why medical physicists are perhaps not as effective as educators as we could be.

To understand the issue of medical physicists as educators, let us review the differences in the education and skills of medical physicists as researchers or clinicians as compared to the education and skills of medical physicists as educators. Once we can recognize these differences, we can then address how to respond to the existence of these differences.

The education that prepares a medical physicist for research or clinical work includes acquisition of core knowledge and skills in advanced physics and mathematics, typically learned during the physicist's undergraduate education, followed by instruction in medical physics principles and medical physics problem solving (thesis), which is typically part of graduate education. Finally, specific clinical skills are learned through either a residency program or on‐the‐job training. The cognitive skills acquired by medical physicists and applied in their practice include analysis, synthesis, and evaluation. These cognitive skills have specific definitions^(1)^. Analysis, for example, is the ability to examine and break information into parts by identifying motives or causes and to make inferences and find evidence to support generalizations. Verbs used to describe analysis include “compare,” “analyze,” “classify,” “distinguish,” “categorize,” “select,” and “prioritize”. Synthesis is the ability to compile information together in a different way by combining elements in a new pattern or proposing alternative solutions. Verbs used to describe synthesis include “compose,” “develop,” “design,” “combine,” “construct,” “produce,” “plan,” and “organize”. Finally, evaluation is the ability to present and defend opinions by making judgments about information, validity of ideas or quality of work based on a set of criteria. Verbs used to describe analysis include “judge,” “relate,” “criticize,” “evaluate,” “critique,” “recommend,” “appraise,” and “compare”.

Analysis, synthesis, and evaluation are all identified as higher‐order cognitive skills, and these activities are what medical physicists are good at, and what differentiates medical physicists from dosimetrists, physics assistants, technologists, etc. Medical physicists achieve these skills through their educational program.

When we look at a medical physicist's educational responsibilities, we find that typically, the medical physicist has had very little formal training. As a consequence, the medical physicist's ability to apply the high‐order cognitive skills of analysis, synthesis, or evaluation to the educational process is limited. Very likely, our teaching skills were obtained by modeling the behavior of our own teachers. Moreover, because we lack understanding of the educational process, we imitate behavior without understanding the rationale for that behavior. That makes us technicians, not scientists. As a consequence we may be reluctant to incorporate new teaching behaviors and our teaching methodologies tend to be conservative.

I would challenge any medical physicist currently involved in education with answering the following two questions:

- How are your present medical physics practice methodologies different from those you used 5 years ago?
- How are your present teaching methodologies different from those you used 5 years ago?

I believe that answering the first question is relatively straightforward. Medical physics is a discipline that is continually in change. I recently proofread a book chapter that I had written about 18 months ago that described some of the procedures in our radiation oncology clinic and found that many of the procedures are now strikingly different from what they were just a short time ago. Yet if I were answer the second question, examine my teaching methodologies, at least those prior to the AAPM Workshop, and compare them to my methodologies from 5 or even 10 years ago, I would have found rather little change. The content of my lectures may have changed to reflect changes in the practice of medical physics, and I was making much more use of the web than I did 5 years ago, but my basic method of teaching had not undergone any significant changes. I do not believe I am very different in that respect from my colleagues.

One way that we as medical physicists could be more amenable to adopting new teaching methodologies and techniques would be for us to learn more about education, to obtain an understanding of the educational process, and to be exposed to various educational theories. The problem with this proposed solution is that the present educational program to prepare medical physicists is already quite extensive, and the addition of more coursework may just prove infeasible without eliminating existing courses, and that's not a solution I would feel comfortable in proposing.

A more practical solution to aid medical physicists in improving teaching abilities, I believe, is for medical physicists to be exposed to various teaching methods through the continuing education process and, even more important, to be willing to adopt new models to our teaching repertoire.

The most valuable time we have in education is the few hours a week we are in contact with our students. The problem is how to make optimal use of this limited time. To do so, we need to modify the role of the teacher of medical physics. We need to view the teacher as a facilitator, rather than as a provider, of information. The teacher provides the student with insight, rather than information. We need to change our approach to the educational process. The transmitting of information, which is currently being done through lecturing, can be done through alternative means, for example, providing our students with recordings of lectures or podcasts. The time with our students should be dedicated to the transmitting of insight, which can be done through techniques such as peer instruction^(2)^ or problem‐based learning^(3)^.

Finally, as a take‐home message on medical physics education, I would like to propose that our role as medical physics teachers is not to teach our students medical physics. Rather, our role as medical physics teachers is to teach our students to learn medical physics.

George Starkschall, PhD

Editor‐in‐Chief

August 15, 2009
